# Preclinical Animal Models for Dravet Syndrome: Seizure Phenotypes, Comorbidities and Drug Screening

**DOI:** 10.3389/fphar.2018.00573

**Published:** 2018-06-04

**Authors:** Aliesha Griffin, Kyla R. Hamling, SoonGweon Hong, Mana Anvar, Luke P. Lee, Scott C. Baraban

**Affiliations:** ^1^Epilepsy Research Laboratory Department of Neurological Surgery and Weill Institute for Neurosciences, University of California, San Francisco, San Francisco, CA, United States; ^2^Department of Bioengineering, University of California, Berkeley, Berkeley, CA, United States

**Keywords:** epilepsy, dravet syndrome, drug discovery, *in vivo*, precision medicine, antiepileptic drugs, animal models

## Abstract

Epilepsy is a common chronic neurological disease affecting almost 3 million people in the United States and 50 million people worldwide. Despite availability of more than two dozen FDA-approved anti-epileptic drugs (AEDs), one-third of patients fail to receive adequate seizure control. Specifically, pediatric genetic epilepsies are often the most severe, debilitating and pharmaco-resistant forms of epilepsy. Epileptic syndromes share a common symptom of unprovoked seizures. While some epilepsies/forms of epilepsy are the result of acquired insults such as head trauma, febrile seizure, or viral infection, others have a genetic basis. The discovery of epilepsy associated genes suggests varied underlying pathologies and opens the door for development of new “personalized” treatment options for each genetic epilepsy. Among these, Dravet syndrome (DS) has received substantial attention for both the pre-clinical and early clinical development of novel therapeutics. Despite these advances, there is no FDA-approved treatment for DS. Over 80% of patients diagnosed with DS carry a *de novo* mutation within the voltage-gated sodium channel gene *SCN1A* and these patients suffer with drug resistant and life-threatening seizures. Here we will review the preclinical animal models for DS featuring inactivation of *SCN1A* (including zebrafish and mice) with an emphasis on seizure phenotypes and behavioral comorbidities. Because many drugs fail somewhere between initial preclinical discovery and clinical trials, it is equally important that we understand how these models respond to known AEDs. As such, we will also review the available literature and recent drug screening efforts using these models with a focus on assay protocols and predictive pharmacological profiles. Validation of these preclinical models is a critical step in our efforts to efficiently discover new therapies for these patients. The behavioral and electrophysiological drug screening assays in zebrafish will be discussed in detail including specific examples from our laboratory using a zebrafish *scn1* mutant and a summary of the nearly 3000 drugs screened to date. As the discovery and development phase rapidly moves from the lab-to-the-clinic for DS, it is hoped that this preclinical strategy offers a platform for how to approach any genetic epilepsy.

## Introduction

While many individuals with epilepsy achieve adequate seizure control with available antiepileptic drugs (AEDs), approximately one-third of patients remain refractory to treatment (Löscher and Schmidt, 2011). With epilepsy being one of the most common chronic neurological diseases worldwide and affecting almost 3 million people in the USA alone, there is a substantial unmet need to identify new AEDs for these refractory epilepsies (Thurman et al., 2011). Epilepsy is clinically defined by the International League Against Epilepsy (ILAE) as “at least two unprovoked (or reflex) seizures” (Scheffer et al., 2017). Although the clinical definition of epilepsy focuses on *unprovoked* seizure activity, the discovery and development of AEDs has traditionally relied, almost exclusively, on preclinical testing using *provoked* seizure models (Wilcox et al., 2013). Provoked seizure models are performed in rodents (wild-type rats or mice) and have led to the successful identification of many clinically useful anti-seizure treatments which often elicit broad-spectrum suppression against a range of different seizure types. However, a significant limitation of these methodologies is that they do not model epilepsy (spontaneous unprovoked seizures). Furthermore, provoked seizure models do not recapitulate the underlying pathology associated with genetic epilepsies. While techniques to genetically modify experimental animals have existed now for several decades, traditional AED discovery programs do not incorporate genetic epilepsy models. Despite the discovery of more than 20 AEDs since phenytoin (Dilantin®) was identified in 1936, the proportion of all patients with drug-resistant epilepsy has remained unchanged (Löscher and Schmidt, 2011), and this is a particularly acute problem for the subset of patients with genetic epilepsies. Therefore, it is worth considering that it may now be time for novel (genetic) models and methodologies for AED discovery.

Recent advances in genomics resulted in tremendous progress in identifying genes associated with epilepsy. Detection of genetic mutations in ion channels, synaptic vesicle proteins, neurotransmitter receptors and transporters, and proteins involved in various metabolic pathways is starting to improve our basic understanding of the varied and complex pathophysiology of epilepsy (Epi4K Consortium; Epilepsy Phenome/Genome Project, 2013; Howard and Baraban, 2017). Moreover, many of the refractory epilepsies previously classified as “idiopathic” can now be explained by single-gene mutations. Identifying the genetic causality of epilepsy also provides an opportunity for a “precision medicine” based approach to developing new treatments. In combination with recent breakthroughs in genome editing technology, there has emerged a unique opportunity for generating and characterizing genetically relevant *in vivo* epilepsy models for the identification and development of disease-specific treatments (Griffin et al., 2016). Particularly for highly intractable genetic epilepsies often seen in children, a disease-specific discovery approach could help identify effective treatments for these patients.

Dravet syndrome (DS) is one such genetic intractable epilepsy. Patients often present with persistent drug resistant seizures within the first year of life. The incidence of DS in the United States is 1 of 15,000–20,000 and almost 80% of patients have a loss-of-function mutation in a single copy of the *SCN1A* gene (Fukuma et al., 2004; Zuberi et al., 2011; Wu et al., 2015). This results in hapolinsuffieiency for the Nav1.1 sodium ion channel and is predicted to be the major pathophysiology resulting in DS (Bechi et al., 2012). As the Nav1.1 sodium channel plays a role in suppressing neuronal excitability, *SCN1A* loss-of-function mutations lead to neuronal hyperactivity and unprovoked seizures. While frequent unprovoked seizures are the main characteristic of epileptic encephalopathies like DS, patients also suffer from a range of comorbidities affecting the areas of locomotion, speech, and behavior. DS patients often have disrupted sleep and metabolic circadian rhythms, neurodevelopmental delay and intellectual disabilities, oculomotor deficits, and psychomotor regression (Nolan et al., 2006; Martin et al., 2010b; Dhamija et al., 2014). Sudden unexpected death associated with epilepsy (SUDEP) is also prevalent in this population (Shmuely et al., 2016). Due to the severity of these comorbidities, effective treatments which can address both seizures and the range of comorbidities associated with DS are urgently needed.

To date, there is no FDA-approved standard of care for DS. There has, at least recently, been an encouraging increase in drug candidates emerging from different preclinical pipelines and progressing into early clinical trials. Assessing the predictive validity of the available preclinical DS models and methodologies is of critical importance when it comes to determining which of these treatments offer the greatest chance of clinical success. This review will focus on the genetic mouse and zebrafish models of DS. Importantly, we will discuss the “construct” (causal mechanism), “face” (phenotypic features), and “predictive” (success in identifying treatments used clinically) validity of these models (and assays).

## Preclinical genetic models of dravet syndrome

Being able to replicate the “construct” or causal mechanism of Nav1.1 deficiency, relies on generating animal models with genetic mutations in the *SCN1A* orthologue gene. Similar to humans, in mice, the sodium ion channel Nav1.1 (*Scn1a*) is expressed throughout the central nervous system including the axon initial segment of parvalbumin-positive hippocampal interneurons and excitatory principal cells (Chen et al., 2004; Ogiwara et al., 2007; Kim et al., 2011). Currently, there are numerous genetic mouse models for DS which aim to replicate *SCN1A* loss-of-function observed in DS. These lines include targeted deletion of *Scn1a* exon 1 (*Scn1a*^tm1Kea^) (Miller et al., 2014) and exon 26 (*Scn1a*^tm1Wac^) (Yu et al., 2006), specific point mutation knock-ins; Scn1a R1407X (Ogiwara et al., 2007), Scn1a R1648H (Martin et al., 2010a), and Scn1a E1099X (Tsai et al., 2015), and a transgenic mouse model expressing a bacterial artificial chromosome (BAC) with the human SCN1A R1648H mutation (Tang et al., 2009). Additionally, several GABAergic neurons conditional knock-out lines have also been generated (Cheah et al., 2012; Dutton et al., 2013; Ogiwara et al., 2013). The development of these genetic mouse models has greatly advanced our understanding of the pathophysiology of DS. For example, several mouse studies demonstrated that haploinsufficiency for Nav1.1 leads to decreased firing in the inhibitory GABAergic interneurons that results in reduced synaptic inhibition, and causing network hyperexcitability and seizures (Yu et al., 2006; Ogiwara et al., 2007; Hedrich et al., 2014).

The best characterized genetic zebrafish model of DS uses a Nav1.1 mutant (*scn1lab*^s552^) first identified in a large-scale chemical mutagenesis screen for larvae with oculomotor deficits conducted by our colleague Herwig Baier (Schoonheim et al., 2010). Zebrafish larvae containing a single nucleotide substitution in the *scn1lab* gene were confirmed to have a loss-of-function in a sodium ion channel with 76% sequence identity to human *SCN1A*. In 2013, it was this *scn1lab*^s552^ mutant zebrafish which we first described as replicating many of the essential clinical phenotypes observed in DS patients (Baraban et al., 2013). Zebrafish have two *scn1a* genes which are both highly expressed within the central nervous system; *scn1lab* and *scn1laa* (Baraban et al., 2013). Genetically, homozygous *scn1lab*^s552^ mutants are haploinsufficient for the Nav1.1 sodium ion channel due to the expression of the duplicated paralogue gene *scn1laa* (67% protein identity to the human Nav1.1). As DS patients are also haploinsufficient for the Nav1.1 sodium ion channel, the zebrafish *scn1lab*^s552^ mutants therefore replicate the genetic etiology observed in the majority of DS patients.

Genomic editing methods such as the CRISPR/Cas9 system (clustered regularly interspaced short palindromic repeat/Cas9) allow for rapid and efficient modification of endogenous genes in a range of animal models (Hwang et al., 2013; Sander and Joung, 2014). Following identification of a potential disease causing allele in patients with epilepsy, it is now plausible to generate models with mutations in the homologous genes. Being able to precisely model patient variants will not only enhance our overall understanding of specific allele pathophysiology but also contribute to patient precision therapies. The development of *in vitro* neuronal models from DS patient derived induced pluripotent stem (iPS) cells or cerebral oganoids also allows for allele specific studies at the molecular and cellular levels (Higurashi et al., 2013; Jiao et al., 2013; Liu et al., 2013, 2016; Sun et al., 2016). The main advantage of patient-derived *in vitro* platforms is that they can precisely model the genetic construct, including any genetic modifiers which may influence disease severity and/or drug response. When evaluating how a given drug alters the function of a voltage-gated ion channel such as SCN1, these reduced systems have significant value. However, these primarily two-dimensional *in vitro* neuronal cultures are unable to recapitulate the full network where these disease causing alleles are embedded, making evaluation of antiepileptic actions for a network disorder like epilepsy difficult, and recapitulating behavioral comorbidities or SUDEP virtually impossible.

Another so-called “simple” species—*Drosophila melanogaster* (fruit flies)—which feature a single voltage-gated sodium channel gene, (*para*) has also been used to model SCN1 mutations. Several *para* mutants have emerged from forward genetic (Siddiqi and Benzer, 1976; Lindsay et al., 2008; Parker et al., 2011) screens or through knock-in of specific disease causing alleles identified in human DS patients (Schutte et al., 2014). Specifically, O'Dowd and colleagues introduced a homologous mutation in the *para* gene designed to replicate the *SCN1A* S1231R identified in a DS patient (Schutte et al., 2014). Similar to DS patients, this mutation resulted in loss-of-function of the sodium channel. While humans have nine different sodium channels, the *Drosphila para* gene produces a range of sodium channels with different functional properties through alternative splicing (Thackeray and Ganetzky, 1994). Many of the *para* mutations reside in an evolutionally conserved constitutively expressed exon and, therefore all the expressed sodium channels are affected. While it is possible to precisely model some patient variants in *Drosophila*, given the difference in genetic and brain architecture between *Drosophila* and humans, many of the molecular, cellular, behavioral and network changes associated with Nav1.1 haploinsufficiency are unable to be replicated in this non-vertebrate model system. Nonetheless, consistent with our discoveries in *scn1a* zebrafish models (discussed in detail below), serotonin pathways were recently implicated as a “potential therapeutic target for DS” based on studies using *SCN1A* S1231R *Drosophila* mutants (Schutte et al., 2014).

## Phenotypic validity of dravet syndrome *in vivo* models

### Detecting spontaneous seizure events in dravet syndrome models

Evaluating epilepsy (spontaneous unprovoked seizures) in genetic models, rather than provoked seizures relies on continuous recording and monitoring efforts. Using video-electroencephalographic (vEEG) monitoring, some form of spontaneous seizure activity has been reported for many of the Nav1.1 haploinsufficient mouse models, though seizure phenotypes (and survival) in these mice is strongly dependent upon background strain owing to strain-specific genetic modifier genes. For example, on the 129S6/SvEvTac background strain *Scn1a*^tm1Kea^ heterozygotes exhibit no overt phenotype and have a normal lifespan. However, when crossed with the C57BL/6J background strain these mice exhibit spontaneous seizures and early lethality around 1 month of age (Miller et al., 2014; Mistry et al., 2014). Similarly, *Scn1a*^tm1Wac^ heterozygotes generated on the 129/SvJ background had no obvious phenotype, but when crossed with the C57BL/6 background strain they develop spontaneous seizures (Yu et al., 2006). These mice die prematurely and have a high incidence of short duration, generalized seizures in the hours preceding death, while prolonged status epilepticus is rare (Kalume et al., 2013). Hyperthermia-induced seizures and SUDEP, have also been extensively documented in *Scn1a* mouse lines (Ogiwara et al., 2007; Oakley et al., 2009; Cheah et al., 2012; Dutton et al., 2013; Kalume et al., 2013). Although febrile seizures are common in the early-life of DS patients, the association between febrile seizures and true epilepsy remains unknown. Furthermore, how seizure phenotypes in these mice respond to available AEDs—discussed in more detail below—has received less attention.

Local field potential (LFP) recording techniques in larval zebrafish offer a reliable method to monitor abnormal electrographic seizure events associated with spontaneous unprovoked seizures. In its simplest form, using a micromanipulator and glass microelectrode patch pipettes with a diameter around 1.2 μm (approximately three times smaller than a single neuron), the larval skin can easily be punctured and a LFP recording can be obtained from any brain structure. In our hands, these recordings are extremely stable for many hours with no diminution of signal quality. To reduce potential twitch-like movement artifacts during LFP recordings, agarose-embedded larvae can be paralyzed with α-bungarotoxin or pancuronium. Importantly, movement artifacts are not multi-spike and have a waveform clearly distinguishable from seizure events. To increase throughput of the electrophysiological assay, we also developed a reliable non-invasive microfluidic platform-based recording approach that uses surface electrodes (thus, some attenuation of signal amplitude with no change in sensitivity) and parallel larvae trapped in individual wells. This integrated Zebrafish Activity Platform (iZAP), developed initially with a 12-well, 5-recording electrodes per fish format allowed us to record brain activity for several hours to days (Hong et al., 2016). An additional advantage of the iZAP system, as it relates to drug discovery, is the ability to wash drugs on, and off, in cross-over style pharmacology studies. Both recording approaches can be used to detect electrographic seizures in larval zebrafish up to 12 days post fertilization (dpf).

The *scn1lab*^s552^ homozygous mutants show spontaneous electrographic seizure activity from 3 dpf. Seizure frequency is highest between 4 and 5 dpf. LFP recordings from paralyzed and agar-immobilized larvae show an ictal-like pattern identified by large-amplitude (>5 times baseline), long-duration (>1,000 ms) events (Baraban et al., 2013). Often frequent, unprovoked small amplitude interictal-like short bursts are also observed at durations between 120 and 300 ms; small amplitude events between 50 and 100 ms in duration are not considered abnormal and can also be routinely observed in wild-type zebrafish between 3 and 10 dpf. Of note, the unprovoked abnormal ictal- and interictal-like electrographic events observed in *scn1lab*^s552^ homozygous mutants are similar in waveform to epileptiform events elicited in wild-type larvae upon exposure to a common convulsant drug, pentylenetetrazole (PTZ) (Baraban et al., 2005; Baraban, 2013) as well as other genetic zebrafish epilepsy models for *stxbp1* (Grone et al., 2017), *mindbomb*/Ube3a (Hortopan et al., 2010), and *aldh19a1* (Pena et al., 2017). Additionally, larvae with a homozygous loss-of-function mutation in the paralogue *scn1laa* gene (*scn1laa*^sa1674^) exhibit spontaneous seizures (Griffin et al., 2017), as does a second N-ethyl-N-nitrosourea (ENU)-generated *scn1lab* mutant (*scn1lab*^sa16474^) (Eimon et al., 2018) supporting the etiology underlying the spontaneous seizures observed in DS patients results from haploinsufficiency of the Nav1.1 sodium ion channel.

As an *in vivo* vertebrate model system, zebrafish larvae also exhibit spontaneous convulsive swim behaviors that are easily monitored using locomotion tracking software at video frame rates of 33 Hz. Based on our initial work with PTZ-induced seizures we established a classification scheme for these larval seizure behaviors: Stage 3 is the most severe, where larvae exhibit high speed (>20 mm/sec), full body convulsions followed by a brief loss of posture for a few seconds; Stage 2 behavior shows increased swim activity rate and a rapid “whirlpool-like” circling; and, slight increase in swim activity (Stage 1) or no swim activity (Stage 0) are considered as normal movement behaviors (Baraban et al., 2005). As an example of a potentially advantageous characteristic for high-throughput assays, PTZ (molecular weight, 367.8 g/mol) at concentrations as low as 2.5 mM elicits the first signs of larval seizure behavior within 5 min of acute exposure suggesting that drug penetrance is rapid and long-term exposures are not necessary. Moreover, high-speed, convulsive, swim behaviors are never observed in wild-type larvae, and at video acquisition rates of 200 Hz and above, these abnormal whole-body seizure behaviors are easily quantified. Based on the frequent high baseline occurrence of high-velocity Stage 3 seizure behaviors and intervening Stage 2 hyperactivity, locomotion plots measuring swim velocity offer a reliable surrogate measure of behavioral seizure activity. This automated locomotion approach serves as the first-stage of our high-throughput drug screening strategy, as described in more detail below.

### Comorbidities and characteristics of dravet syndrome animal models

DS is a multifaceted disease and thus there is a demand to understand the serious and complex comorbidities often experienced by patients. Most patients with DS have intellectual disability, and experience other neurodevelopmental disorders including autism from the second year of life. While it is expected that laboratory animals cannot recapitulate the full spectrum of human behaviors or cognition (and this is a limitation of all experimental model systems), it is encouraging that some of these comorbidities can be modeled at the preclinical level in *Scn1a* mutant mice. For example, mice with the *Scn1a*^tm1.1Kzy^ loss-of-function allele (Scn1a R1407X), showed hyperactivity, altered anxiety-like behavior, lowered sociability, lack of social novelty preference, and spatial learning and memory impairment (Ito et al., 2013). Furthermore, many of these behavioral deficits were reported in mice with conditional deletion of *Scn1a* in parvalbumin-positive interneurons (Tatsukawa et al., 2018), suggesting some comorbid behaviors in DS patients are mediated specifically by this interneuron sub-population. Simmilarly, the Scn1a^tm1Wac^ heterozygote mice display hyperactivity, anxiety-like behavior, increased stereotypies, impaired social behavior, and impairment spatial learning and memory (Han et al., 2012). Additionally, conditional deletion of Scn1a in forebrain GABAergic neurons recapitulates many of the autism-related phenotypes and spatial learning deficits highlighting a putative role for interneuron dysfunction in these comorbid behaviors (Han et al., 2012). Importantly, treatment with low-dose clonazepam (Han et al., 2012), a benzodiazepine and GABA agonist, was shown to completely rescue many of the behavioral deficits indicating pharmacological treatment could improve not only seizures but also some of the comorbid behaviors observed. Subsequently, these mouse studies highlight the potential to assess the effectiveness of treatments against the comorbidities experienced by DS patients.

Sleep disturbance is often reported in DS patients and is more prevalent than levels reported in young children in the general population and general epilepsy cohorts (Licheni et al., 2018). Mimicking sleep disturbances reported in DS patients, abnormalities in sleep behavior and circadian rhythms have been observed in several Nav1.1 deficient mouse models. In mice, Nav1.1 is expressed in the regions of the brain known to regulate sleep and circadian rhythms (Han et al., 2012; Papale et al., 2013). The *Scn1*a^tm1Wac^ heterozygotes show longer circadian period, with delayed onset of activity during the dark phase (Han et al., 2012). Combined pharmacological treatment with tiagabine and clonazepam was able to rescue the impaired circadian behavior without inducing sedative effects, highlighting again the potential for *Scn1a* mouse models in assessing compounds against specific DS comorbidities (Han et al., 2012). Finally, several of the *Scn1a* mouse knockout and knockin lines exhibit premature death (Yu et al., 2006; Cheah et al., 2012; Auerbach et al., 2013; Kalume et al., 2013), which offer an important research tool for understanding mechanisms of SUDEP. The sodium channel blocker GS967, was shown to improve the survival of the *Scn1a*^tm1Kea^ x C57BL/6J F1 mouse model (Anderson et al., 2017) despite sodium channel blockers being contraindicated for most DS patients. A distinct advantage of rodent *Scn1a* knockout models lies in the range of complex bahaviorial phenotypes that can be assayed. Significant clinical benefit on one, or more, of these comorbid behaviors could help differentiate between novel antiepileptic drug candidates currently under evaluation for DS.

Although modeling complex neurological behaviors in larval zebrafish is also limited, some behavioral comorbid characteristics observed in DS patients can be recapitulated. Like mice haploinsufficient for Nav1.1, the *scn1lab*^s552^ homozygous mutants also exhibit premature death and fail to thrive past 12 dpf. Additionally, *scn1lab*^s552^ homozygous mutants have night-time hyperactivity, suggesting disrupted sleep behaviors and diurnal locomotor activity deficit similar to what is observed in DS patients and mouse models (Grone et al., 2017). Furthermore, DS zebrafish larvae show “wall hugging” behavior (thigmotaxis) which is often considered a sign of increased anxiety in larvae (Schnörr et al., 2012; Baraban et al., 2013; Grone et al., 2017). Interestingly, the *scn1lab*^s552^ homozygous mutant zebrafish was first characterized as having saccadic eye movement abnormalities, a characteristic often found in pediatric epilepsies and DS patients (Lunn et al., 2016). Equally, altered glycolytic metabolism and oxygen consumption rates are a characteristic deficit in many pediatric epileptic syndromes and were shown in novel metabolic larval zebrafish assays first described by Kumar et al. (2016) to be altered in *scn1lab*^s552^ homozygous mutants. Importantly, the ketogenic diet, a treatment which has reported efficacy in some DS patients (Dressler et al., 2015), returned the altered metabolism of the larvae to normal levels and significantly suppressed seizures (Baraban et al., 2013; Kumar et al., 2016).

As the repertoire of complex zebrafish larvae behaviors become better understood, researchers can focus on clinically relevant characteristics and comorbidities as additional measurable outcomes for drug discovery. Pharmacological studies investigating the effect of diazepam, valproate, trazodone and clemizole on *scn1lab*^s552^ homozygous mutant behaviors was recently reported by our laboratory (Grone et al., 2017). Results showed that clemizole and diazepam reduce the nighttime hyperactivity and decrease the anxiety-like wall hugging-behavior observed in *scn1lab*^s552^ homozygous mutants to control levels. Consistent with these findings, diazepam is often prescribed for anxiety and short-term insomnia in humans. Conversely, the antidepressant trazodone which is frequently prescribed off-label for sleep issues (Wong et al., 2017) showed no effect on improving nighttime hyperactivity deficits in *scn1lab*^s552^ homozygous mutants (Grone et al., 2017). As the impact and severity of comorbidities of DS becomes better understood, animal models with good “face” validity, i.e., recapitulating the spontaneous seizures and comorbid characteristics, offer valuable preclinical tools to improve clinical treatments for patients.

## Assessing the predictive validity of DS animal models

The ILAE definition of epilepsy includes a distinction for unprovoked seizures (Scheffer et al., 2017). However, traditional studies of epilepsy, including all of the legacy and current rodent models offered by the NIH Epilepsy Therapy Screening Program (ETSP) (https://www.ninds.nih.gov/Current-Research/Focus-Research/Focus-Epilepsy/ETSP) involve induced or provoked seizures: maximal electroshock test (MES), metrazol seizure threshold (MET), 6 Hz 44 mA seizure model, corneal kindled seizure model, lamotrigine-resistant amygdala kindled seizure model, and mesial temporal lobe epilepsy (mTLE) induced by focal chemoconvulsant injection. These are considered models of generalized tonic-clonic seizures, temporal lobe epilepsy or clonic seizures but *do not* model any genetic form of epilepsy. While this approach has successfully identified new anti-seizure drugs, the fact that 30–40% of all epilepsy patients remain resistant to available AEDs discovered using these ETSP models also suggests different pharmacological efficacy between spontaneous unprovoked seizures (epilepsy) and seizures that are induced/provoked. Furthermore, it suggests that these traditional seizure inducible rodent models are not appropriate as animal models for intractable genetic epilepsies. In fact, AED discovery guided by these ETSP preclinical models, does not incorporate any model of DS or other genetic human epilepsy in its repertoire. This may explain why DS remains refractory to drugs identified through this program and suggests limited predictive validity of these inducible seizure models when determining clinical efficacy for DS patients. In contrast, genetic mouse and/or zebrafish models exhibiting epilepsy (unprovoked seizures) offer a valid alternative, but currently under-appreciated (Galanopoulou et al., 2012; Simonato et al., 2014, 2017), approach for preclinical drug discovery and development. It is our strong opinion that genetic models which faithfully recapitulate clinical phenotypes (i.e., “construct” validity) and characterized by spontaneous unprovoked seizures (i.e., “face” validity) offer the most appropriate preclinical pathway to new drug discovery. To assess the “predictive” validity of each genetic model, or assay, two factors need to be considered as a rigorous form of model validation: (i) whether the seizures are unprovoked and spontaneous in origin, and (ii) whether the pharmacological responses represent what is observed in DS patients.

Genetic models of epilepsy that show clinically relevant phenotypes should exhibit the same pharmacological profile as DS patients as a form of “predictive” validity. Although there is no FDA-approved treatment there is a clinical “standard of care” for DS which advises a recommended AED polytherapy for most patients. Retrospective studies of AED responses of DS patients rank benzodiazepines (clobazam, diazepam), valproate, or stiripentol as the most effective options and are considered as the standard of care “first line” treatment for most of this patient population (Hawkins et al., 2017; Villas et al., 2017). Additionally, topiramate, potassium bromide and the ketogenic diet have also shown some efficacy for DS (Caraballo, 2011; Wirrell, 2016; Villas et al., 2017; Lagae et al., 2018). At the same time, DS also meets the ILAE classification for a drug resistant epilepsy i.e., “failure of adequate trials of two tolerated and appropriately chosen and used AED schedules (whether as monotherapies or in combination) to achieve sustained seizure freedom” (Kwan et al., 2010). Finally, a number of AEDs are contraindicated and known to exacerbate seizures in DS including, carbamazepine, oxcarbazepine, and lamotrigine. Given the wide range of *de novo* mutations associated with DS it is also not surprising that some patients exhibit atypical experiences with sodium channel blockers, levetiracetam or other AEDs not recommended as the standard of care (Genton et al., 2000; Snoeijen-Schouwenaars et al., 2015; Takaori et al., 2017). Based on these clinical observations, a validated animal model of DS should identify AEDs commonly used in a clinical setting, while at the same time demonstrating a failure to control seizures with at least two or more appropriate AEDs.

Currently, there is no rodent model of DS which has been pharmacologically validated against epilepsy i.e., spontaneous unprovoked seizures. While this is technically possible, the logistics of capturing a sufficient number of unprovoked seizure events to adequately power a statistical analysis of a given drug treatment would require hundreds of hours of labor-intensive continuous vEEG monitoring across many animals, potentially taking months (to years) for adequate analysis of even a handful of drug candidates. The incomplete penetrance, low seizure frequency and early fatality observed in *Scn1a* mutant mouse strains are additional confounds that render mouse models as a less than ideal for predicating efficacy of AEDs against spontaneous seizures. Recently, Kearney and colleagues attempted to pharmacologically validate the more severe *Scn1a*^tm1Kea^ x C57BL/6J F1 mouse line in this manner (Hawkins et al., 2017). In evaluating four AEDs including clobazam, valproate, and topiramate against spontaneous seizures, this model failed to show efficacy in suppressing seizures for any of these “first line” DS drugs. Lamotrigine produced a significant elevation of spontaneous seizure frequency in this model, consistent with what is observed in patients, and three additional AEDs failed to suppress seizures consistent with a drug resistant epilepsy classification (Table 1).

**Table 1:** Predictive validity of DS models.

	***Antiepileptic activity***						
**AED**	**DS patients**	***scn1lab***^s552^ **zebrafish**	***scn1lab***^s552^ **zebrafish**	***scn1lab*** **MO zebrafish**	**DS mice**		
		**Spontaneous (acute)**	**Light induced**	**Spontaneous (long-term)**	**Hyperthermia-induced**	**Spontaneous**	**Spontaneous (primed)**
Valproate	Yes^a^^,^^b^^,^^c^	Yes^d^^,^^e^		Yes^j^	Yes^b^	No^b^	No^b^
Clobazam	Yes^a^^,^^b^^,^^c^		No^i^		Yes^b^	No^b^	Yes^b^
Stiripentol	Yes^a^^,^^b^^,^^c^	Yes^d^	No^i^	No^j^	No^b^		No^b^
Topiramate	Yes^a^^,^^b^^,^^c^	Yes^e^			No^b^	No^b^	
Clonazepam	Yes^a^^,^^b^^,^^c^				Yes^k^		
Bromides	Yes^a^^,^^c^	Yes^d^					
Diazepam	Yes^a^	Yes^d^	No^i^				
Levetiracetam	No^a^^,^^b^	No^f^^,^^h^			Yes^b^	No^b^	
Ethosuximide	No^a^^,^^c^	No^d^^,^^h^					
Zonisamide	No^a^^,^^b^	No^f^					
Carbamazepine	No (worse)^a^^,^^c^	No^d^^,^^f^^,^^g^^,^^h^	No^i^	No^j^	No^b^	No^b^	
Phenytoin	No (worse)^a^^,^^b^^,^^c^	No^f^^,^^g^^,^^h^			No^b^		
Lamotrigine	No (worse)^a^^,^^b^^,^^c^	No^f^^,^^g^^,^^h^			No^b^	No^b^	
Oxcarbazepine	No (worse)^a^^,^^b^	No^f^^,^^g^^,^^h^					
Phenobarbital	No^a^^,^^b^^,^^c^				Yes^b^	No^b^	
Gabapentin	No^a^	No^h^					
Rufinamide	No^a^	No^f^^,^^g^					
No. of AEDs predictive		14/14	1/4	2/3	5/9	4/7	1/3

a (Villas et al., 2017),

b (Hawkins et al., 2017),

c (Chiron, 2011);

d (Baraban et al., 2013);

e Hong et al. (2016),

f see Figure 3,

g (Griffin et al., 2017),

h (Dinday and Baraban, 2015),

i (Eimon et al., 2018),

j (Zhang et al., 2015),

k *(Oakley et al., 2013)*.

Due to the lower seizure frequency and variable phenotypes observed in Nav1.1 deficient mice, there is a growing trend to use traditional inducible seizure methods layered on the *Scn1a*^+/−^ mouse background. Given the discrepancy between data on provoked versus unprovoked seizures models, simply inducing seizures in an animal with a Nav1.1 deficiency comes with the risk of identifying compounds which impact the seizure-induction mechanism and will ultimately not be effective against spontaneous seizures in DS patients. Oakley et al. (2013) reported the AED clonazepam was effective in suppressing hyperthermia-induced seizures in the *Scn1a*^tm1Wac^ C57Bl/6 line, but it also increased motor impairment at therapeutic doses. When evaluating the pharmacological validity of several AEDs to suppress hyperthermia-induced seizures, the *Scn1a*^tm1Kea^ × C57BL/6J F1 mouse mouse model succeeded in identification of valproate and clobazam but failed to identify topiramate and stiripentol (Hawkins et al., 2017). Additionally, phenobarbital, a drug with limited efficacy reported in DS patients was identified as a false positive. The limited accuracy of this pharmacological validation also makes it difficult to interpret recent reports on huperzine A or cannabidiol in *Scn1a* mice where only protection against provoked hyperthermia-induced seizures were described (Wong et al., 2016; Kaplan et al., 2017). For example, huperzine A suppressed seizure activity in a hyperthermia-induced assay in *Scn1a*^R1648H/+^ mice and a PTZ assay in wild-type mice or zebrafish (Wong et al., 2016), but failed to suppress *scn1lab*^s552^ spontaneous seizures when tested directly (Dinday and Baraban, 2015), or screened blindly as part of a natural products drug library (see Figure 3D). As further evidence for a discrepancy between drugs that block hyperthermia-induced seizures versus those effective against spontaneous seizures, we reported a powerful suppression of hyperthermia-induced electrographic seizure events in a zebrafish model (Hunt et al., 2012) with NMDA receptor blockers (ifenprodil and MK-801) but neither drug inhibited spontaneous electrographic seizure events in *scn1lab*^s552^ mutant zebrafish; MK-801 actually increased spontaneous seizure frequency (Dinday and Baraban, 2015; Griffin et al., 2017). Taken together, these mixed results suggest that there is limited predictability in DS mouse models and therefore *Scn1a* mutant mice may not be the first (or even second) choice to screen existing or experimental AEDs.

Zebrafish larvae offer an alternate *in vivo* model for the assessment anti-seizure efficacy of AEDs for DS. While this model may not fully recapitulate complex cognitive and neurobehavioral abilities, as an epilepsy model it offers a valid alternative. In our initial description of *scn1lab*^s552^ mutant zebrafish, we not only characterized the epilepsy (unprovoked seizure) phenotype, but also established a reliable acute exposure assay to validate this model. Briefly, we successfully demonstrated the anti-seizure properties of available AEDs in a two-stage assay: (i) high-throughput automated video behavior tracking of individual larvae to identify drugs which reduce high speed swim behavior (Stage 2 and Stage 3) associated with an epilepsy phenotype to control (Stage 0 or 1) levels, and (ii) lower-throughput electrophysiological assessment to confirm drug suppression of spontaneously occurring electrographic seizure events within the brain. As abnormal electrical events in the brain are the hallmark of epilepsy, individual LFP recordings are essential to exclude false positive hits from the behavior assay (such as sedatives or muscle relaxants). More importantly, we do not advocate using the behavioral assay as a stand-alone screening tool in the absence of electrophysiological confirmation.

Using this two-stage assay, incorporating behavioral and electrophysiological techniques pioneered for zebrafish larvae over the past decade in our laboratory, we showed suppression of unprovoked seizure activity with AEDs (valproate, diazepam, stiripentol, potassium bromide and topiramate) prescribed for DS patients (Table 1). Furthermore, *scn1lab*^s552^ mutant zebrafish also fail to respond to eight different AEDs which are contraindicated for DS including ion channel inhibitors like carbamazepine or ethosuximide. Using *scn1lab*^s552^ homozygous mutant larvae at 5 dpf larvae ensures a high frequency of baseline spontaneous seizures, allowing us to use relatively short assay times (30 min), generate high statistical power and maintain high screening throughput. Moreover, each treated larva is normalized to its own baseline to account for inherent differences often observed during *in vivo* behavioral assessments. Using this two-stage approach, we successfully demonstrated that the pharmacological profile of the *scn1lab*^s552^ mutants resembles that of DS patients with 100% accuracy (i.e., 14 of 14 AEDs correctly classified; Table 1). The predictive validity accuracy of this model and assay confers confidence in our ability to identify novel drug candidates using *scn1lab*^s552^ zebrafish.

In contrast to our published studies (Baraban et al., 2013; Dinday and Baraban, 2015; Griffin et al., 2017), others have trialed long-term protocols with lower drug concentrations and longer drug exposures (Zhang et al., 2015; Sourbron et al., 2016, 2017a). Initial long-term exposure studies at 24- and 48-h utilized a morpholino-based *scn1lab* knock-down approach and screened five known AEDs against spontaneous behavioral seizures. While valproate, clobazam, topiramate and stiripentol were shown to be effective in the behavioral seizure assays, only valproate was established to suppress electrical seizure activity in the brain, confirming the successful predictive validation for only one AED typically used in DS (Table 1). Given the transient knockdown observed by morpholinos, the high variability in the swim behavior observed in control larvae (Zhang et al., 2015), concerns about off-target effects, particularly neuronal defects and differences observed in knockdown verse knockout approaches reported in the zebrafish community (Stainier et al., 2017), it is difficult to envision how this approach can be reliably used for large-scale screening efforts.

**Figure 1:**
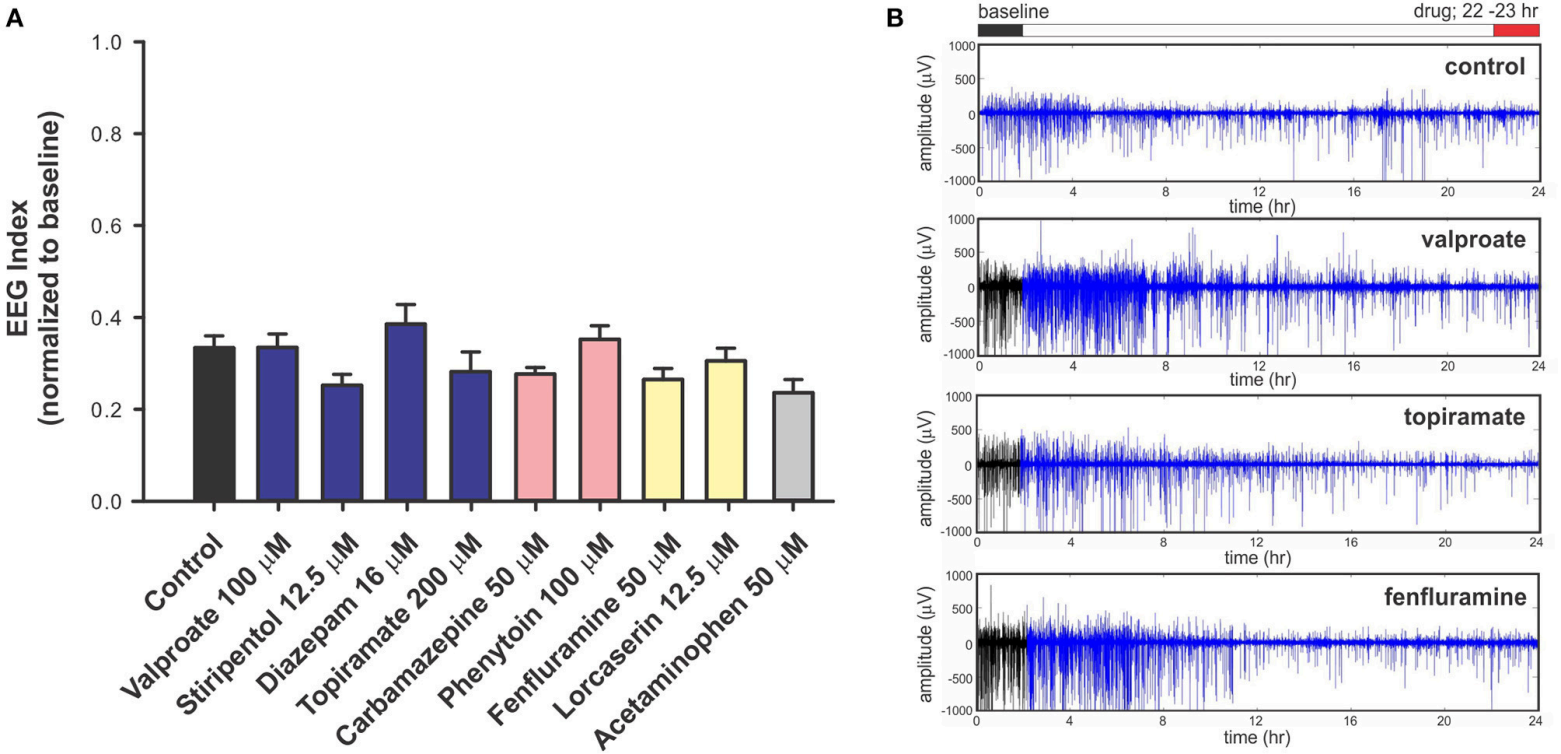
Electrophysiology analysis of *scn1lab*^s552^ homozygous mutant larvae during long-term exposure of AEDs. For non-invasive long-term monitoring of electrographic seizure activity in larval zebrafish, we used an integrated zebrafish activity platform (iZAP) microfluidic recording system previously described by our laboratory (Hong et al., 2016). The iZAP system consists of three primary components: (i) microfluidic unit, (ii) multi-electrode array, and (iii) integrated electronic unit with multichannel amplifiers for simultaneous monitoring of surface EEG activity on five independent electrodes for up to 12 larvae at an acquisition rate of 1 kHz. For these experiment, *scn1lab*^s552^ homozygous mutants screened from larval clutches based on pigmentation are loaded into the iZAP at 5 dpf and continuously monitored for 24-h in embryo medium (or test drug) supplemented with 300 μM pancuronium. A custom algorithm based on distinct features of the zebrafish electrographic seizure signal and spatial correlation between the 5 surface electrodes was used to detect and score seizure events, as described (Hong et al., 2016). For each test compound, we first obtained a 2-h baseline recording, followed by a 22-h drug exposure; at least seven larvae were tested per drug/per experiment and all experiments included at least one biological replicate. Recordings were normalized to the baseline seizure activity and presented as an EEG index where a ratio of 1.0 would represent no change in activity at 22-h vs. baseline and 0.0 would represent a complete suppression of seizure activity. **(A)** Plot of all drug treatments and controls (embryo media) tested in the iZAP device using the low micromolar concentrations published in Sourbron et al. (2016), Sourbron et al. (2017b), or Zhang et al. (2015). Recommended treatments (blue), contra-indicated treatments (red), and recent experimental treatments (yellow) for DS are shown; acetaminophen is also shown (gray). Note that electrographic seizure activity diminishes by approximately 60% from baseline in all *scn1lab*^s552^ homozygous mutants larvae with no significant differences noted between putative antiepileptic drugs, control and acetaminophen exposures using these low micromolar, 22-h drug exposures (Sourbron et al., 2016, 2017a). Graph represents mean (±SD). Statistical significance was determined by one-way ANOVA followed by Dunnet's multiple comparison test. **(B)** Representative raw EEG recordings from one surface electrode channel (of five) are shown for the entire 24-h recording period. Baseline recordings are indicated in black; drug exposures are shown in blue. These raw traces highlight the reduction in electrographic seizure activity seen over time under any recording condition at this stage of *scn1lab*^*s*552^ larval development. In contrast to these data, but consistent with our acute LFP recording protocol, we previously used the iZAP recording device to demonstrate a significant and reversible suppression of electrographic activity monitored in *scn1lab*^s552^ homozygous mutants (2-h drug exposure epochs) for valproate (1 mM), topiramate (1 mM), lorcaserin (250 μM) and trazodone (250 μM) (Hong et al., 2016; Griffin et al., 2017) further highlighting the discrepancy between acute and chronic drug assays.

**Figure 2:**
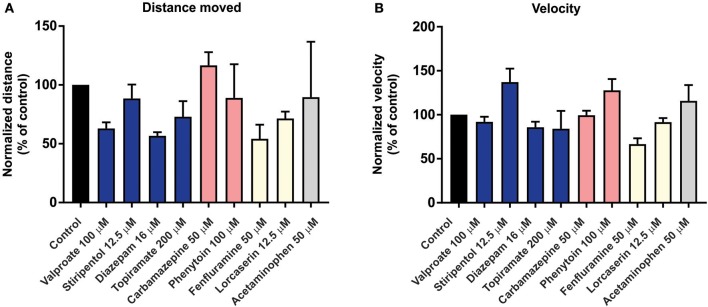
Behavior analysis of *scn1lab*^s552^ homozygous mutant larvae during long-term exposure of AEDs. To examine the effect of long-term exposure of AEDs on *scn1lab*^s552^ swim behavior was analyzed using the DanioVision running EthoVision XT software (DanioVision, Noldus Information Technology). As previously described (Sourbron et al., 2017b), 6 dpf *scn1lab*^s552^ homozygous mutants were identified based on pigmentation and arrayed in a 96-well plate. Larvae were treated with 2% DMSO (control) or compound using drug concentrations previously published (Zhang et al., 2015; Sourbron et al., 2016, 2017a). After 22-h incubation at 28°C with a 14:10 h light/dark photoperiod, and 30 min of chamber habituation, the swim behavior of 7 dpf larvae was analyzed for 10 min under dark conditions. Both the **(A)** total distance moved and **(B)** velocity were analyzed by normalizing the activity of AED treated larvae to vehicle treated controls (previously described as method A, Sourbron et al., 2017b). Graphs represents mean (±SD) normalized to control treated larvae from three independent experiments using the average from 12 larvae per treatment each time. Statistical significance was determined by one-way ANOVA followed by Dunnet's multiple comparison test. No statistical significance changes in swim behavior of *scn1lab*^s552^ homozygous mutants when exposed to known AEDs or putative AEDs. Recommended treatments (blue), contraindicated treatments (red) and recent experimental treatments (yellow) for DS are shown. Acetaminophen is also shown (gray). These behavioral results, which also fail to distinguish between any of the experimental situations tested, are entirely consistent with the data independently obtained using the iZAP system and fail to support the validation of this low micromolar, 22-h exposure assay as an effective means to identify drugs with antiepileptic activity in *scn1lab*^s552^ homozygous mutants.

To investigate the predictive validity of long-term exposure protocols, we initiated a series of behavioral and electrophysiological experiments to test AEDs using *scn1lab*^s552^ zebrafish in the same low micromolar drug concentrations described by de Witte and Lagae (Sourbron et al., 2016, 2017a). Using the iZAP multi-fluidic recording device for non-invasive continuous uninterrupted monitoring of EEG activity of zebrafish larvae (Hong et al., 2016), we screened known and putative AEDs as described (Zhang et al., 2015; Sourbron et al., 2016, 2017a). We previously demonstrated the efficiency of the iZAP device to continuously record EEG data from up to 12 larvae simultaneously during baseline, drug exposure and washout; valproate and topiramate were published as examples of the validity of this recording device to identify clinically relevant AEDs with the ability to suppress unprovoked electrographic seizure activity in *scn1lab*^s552^ zebrafish (Hong et al., 2016). Using the long-term exposure protocol, we consistently observed an approximately 60% decrease in electrographic seizure activity (compared to baseline) at the 22-h timepoint in vehicle-exposed zebrafish. This reduction in unprovoked seizure activity was identical to that seen at the 22-h timepoint with a control drug exposure that has no known antiepileptic activities (acetaminophen). This confirms that the seizure frequency of *scn1lab*^s552^ zebrafish larvae naturally decreases after 5 dpf (regardless of treatment) which is consistent with previously published electrophysiology recordings (Baraban et al., 2013; Hong et al., 2016). Most importantly, in our hands, this decline in spontaneous EEG seizure activity was noted with every drug tested, using concentrations and long-term drug periods identical to those described (Zhang et al., 2015; Sourbron et al., 2016, 2017a; Figure 1). Because we could not distinguish between the effects of acetaminophen (or vehicle) and AEDs known to inhibit electrographic seizures in *scn1lab*^s552^ DS zebrafish (valproate, stiripentol, diazepam), AEDs contra-indicated for DS (phenytoin, carbamazepine) or candidate drugs (fenfluramine and lorcaserin) using the low micromolar concentration, 22-h long-term exposure protocol we conclude that these assays are not valid predictors of antiepileptic drugs. In an additional attempt to validate these long-term protocols, we performed similar studies using our DanioVision locomotion tracking assays and again observed a natural decrease in spontaneous seizure behavior at 22-h, and a failure to distinguish between controls, AEDs or candidate drugs (Figure 2). Our inability to replicate the low micromolar concentration, 22-h exposure assay data using the same *scn1a* zebrafish model suggests that assay (and not necessarily model) differences can account for discrepancies in the literature. Taken together, our results indicate that (i) “positive hits” identified in long-term exposure assays (albeit using a validated *scn1lab*^s552^ DS zebrafish line) should be interpreted with caution and (ii) acute versus long duration incubations of larvae with test drugs can make a substantial difference in outcome.

## Phenotypic drug screening for dravet syndrome

Whole organism phenotypic drug screening provides an unbiased approach to systematically identify molecules that can modify a specific disease phenotype. Although mice offer strengths for understanding the basic biology and pathophysiology of epilepsy, they are not well suited to higher throughput drug screening platforms. In contrast, many aspects of zebrafish biology make them amenable for moderate- to high-throughput drug screening (i) unlike rodents, zebrafish larvae are not fetal but are closer to a “juvenile” state in that the nervous system is mature, vital organs are functioning and tissue architecture is fully developed within the first few days post-fertilization; (ii) only milligrams of compound are needed for screening in 96-well plates as larvae; (iii) zebrafish are reasonably tolerant to dimethylsulphoxide (DMSO) concentrations generally used in drug libraries; and (iv) small molecule compounds dissolved in the swimming medium reach larval target tissues via rapid diffusion through the skin.

Using a whole-animal approach is also advantageous for identifying compounds targeting a network disorder such as epilepsy, as the complex neuronal interactions, vascular components, and neurotransmitter signaling pathways are difficult to recapitulate *in vitro*. Larval zebrafish enable parallel screening for toxicity and activity within the central nervous system, a critical requisite for targeting diseases that affect the brain. Given the evidence that the zebrafish *scn1lab*^s552^ model recapitulates salient genetic, behavioral, electrophysiological phenotypes observed in DS patients, and has been pharmacological validated against known AEDs, this model appears ideal for screening compound libraries for antiepileptic activity (with appropriate validated assays). Furthermore, because *scn1a* homozygous mutant zebrafish exhibit a very high baseline seizure frequency—approximately one ictal-like electrographic seizure event per minute with up to one interictal-like event per second—even relatively short recording epochs (10–20 min) are more than sufficient to monitor seizure activity and power statistical studies for drug discovery.

**Figure 3:**
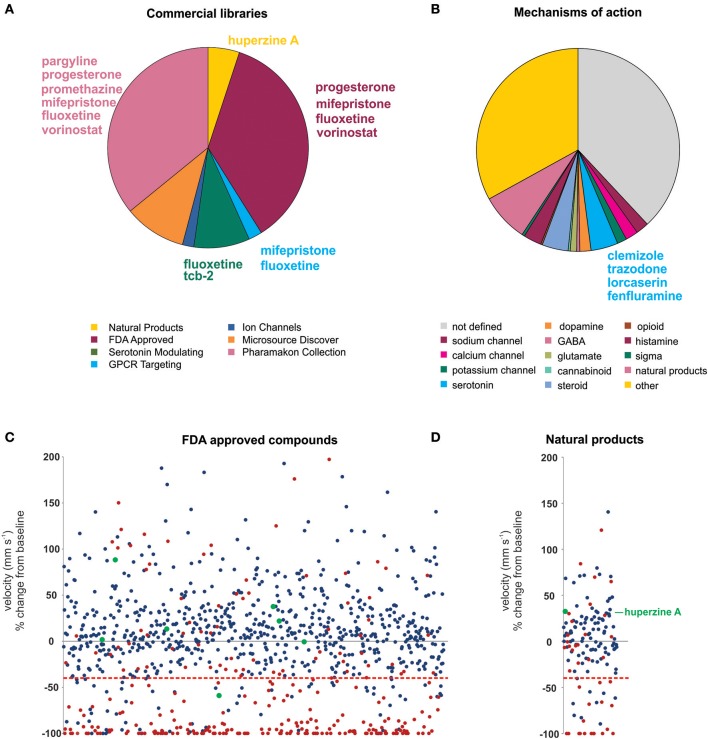
Summary of the compound screening results using the *scn1lab*^s552^ homozygous mutant larvae to identify drugs for DS. Using our pharmacologically validated dual-stage assay screening protocol, 2,863 compounds have been blind tested for anti-seizure activity in the *scn1lab*^s552^ homozygous zebrafish larvae. **(A)** Seven commercially sourced drug libraries were screened including the MicroSource Discovery Systems' International Drug Collection (Baraban et al., 2013) and Pharmakon Collection (Dinday and Baraban, 2015) and Selleckchem's, ion channel library, GPCR compound library, a serotonin modulating compound library (Griffin et al., 2017), a natural product library and a FDA-approved compound library. Compounds highlighted in this plot were reported by other groups as being effective anti-seizure compounds, but could not be confirmed as such in our hands. Specifically, these compounds failed to induce an antiepileptic response when screened blinded as part of these commercial libraries. Blind screening of libraries allows for unbiased testing of compounds regardless of their mechanism of action. **(B)** A summary of identified mechanism of actions of all compounds screened highlighting the broad range of mechanisms covered by these libraries. Clemizole, trazodone and lorcaserin effectively suppressed seizure activity in the *scn1lab*^s552^ homozygous zebrafish larvae. These compounds have known activity at serotonin 2 receptors. 4.3% of all drugs tested are known to modulate serotonin signaling, however, only these drugs were effective. The serotonin reuptake inhibitor fenfluramine is also effective in suppressing *scn1lab*^s552^ homozygous larvae seizure activity and is currently in clinical trial for DS. **(C)** The FDA approved compound library, and **(D)** natural product library were also screened for compounds inhibiting seizure activity. Plots show the locomotor seizure behavior for 5 dpf *scn1lab*^s552^ mutants during the first stage screening. The threshold for inhibition of seizure activity (positive hits) was determined as a reduction in mean swim velocity of 40% (red line). Red data points represent compounds that were classified as toxic as treated larvae have no visible heartbeat or movement in response to touch after 90 min exposure. Green data points represent known AEDs. The natural product huperzine A which has been shown to be effective against hyperthermia induced seizures is labeled. No additional lead compounds were identified in these libraries.

**Figure 4:**
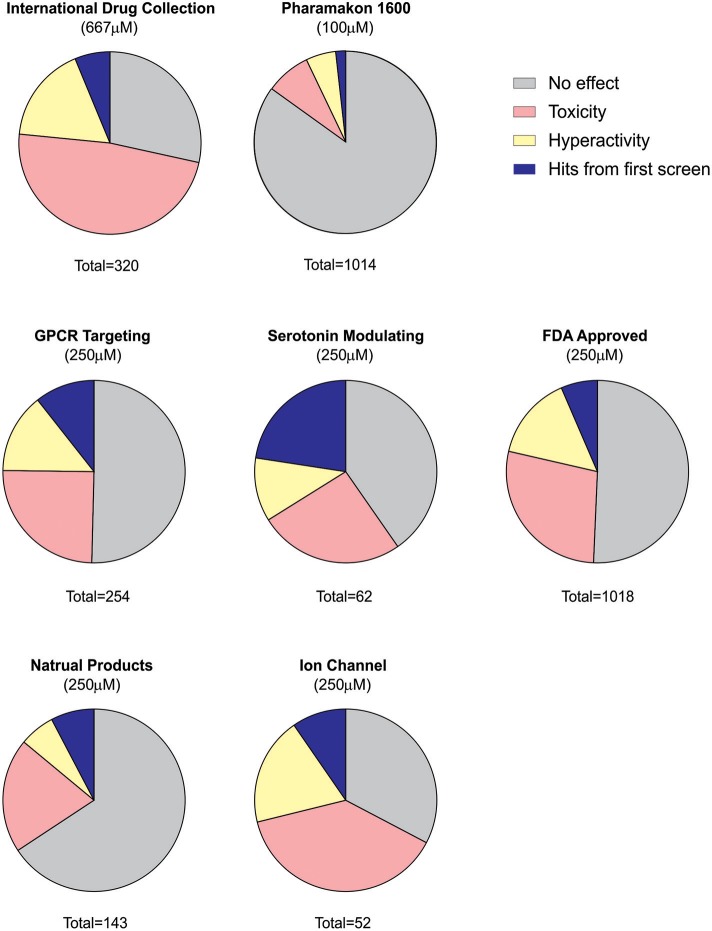
Summary of behavioral screening results for anti-seizure compounds in DS zebrafish larvae. In total, seven commercially available libraries have undergone blind screening for compounds which suppress the seizure activity in *scn1lab*^s552^ homozygous larvae. These include the MicroSource Discovery Systems' International Drug Collection (Baraban et al., 2013) and Pharmakon Collection (Dinday and Baraban, 2015) and Selleckchem's, ion channel library, GPCR compound library, a serotonin modulating compound library (Griffin et al., 2017), a natural product library and a FDA-approved compound library. Plots represent the results from the first blinded screening of each library. A hit is recognized as a compound which reduces the high-speed seizure like swim behavior by more than 40% (>2 S.D.). Once a hit is retested it is then screened by an independent laboratory member. If identified as a hit again, the compound is unblinded and independently sourced for further testing including the second stage electrophysiology assay. The serotonin library exhibited the greatest percentage of positive hits in the first-pass behavioral assay consistent with subsequent identification of four serotonin modulating drugs for the potential treatment of DS e.g., clemizole, trazodone, lorcaserin and fenfluramine. It is also interesting to note that the percentage of toxic drugs is greatest in the ion channel library cohort. Approximately 20% of compounds are identified as toxic when screened at 250 μM as they result in decreased or absent heart beat and/or an absent touch-evoked escape response after 90 min of exposure. When screening at 667 μM, 48% of compounds were identified as toxic. The majority of compounds fail to elicit any significant change in the swim velocity of the larvae.

Using an acute exposure protocol and a two-stage screening platform the Baraban laboratory has screened seven commercially-available libraries consisting of almost 3000 compounds spanning multiple drug classes and targeting several suggested therapeutic mechanisms (Baraban et al., 2013; Dinday and Baraban, 2015; Griffin et al., 2017; Figure 3). After repeated locomotion testing, including assaying independently sourced compounds, only 13 compounds (<0.5%) have been identified by electrophysiology as “false positive” hits. These include the anesthetic lidocaine, the muscle relaxant pancuronium bromide, the N-methyl-D-aspartate antagonist MK-801 and the hallucinogen TCB-2 which was previously identified as an anti-seizure drug. (Sourbron et al., 2017a). On average, approximately 20% of compounds are classified as toxic when screened at 250 μM as they result in decreased or absent heart beat and/or an absent touch-evoked escape response after 90 min of exposure. Screening at higher (667 μM; Baraban et al., 2013) or lower (100 μM; Dinday and Baraban, 2015) concentrations as the assays were optimized, we observed toxicities of ~50% and <10% respectively (Figure 4).

Probably the greatest advantage of zebrafish assays is that they facilitate blinded phenotypic screening of compound libraries and unbiased discovery of new AED candidates. For example, clemizole was identified to exert a powerful suppression of behavioral and electrographic seizures in the *scn1lab*^s552^ larvae (Baraban et al., 2013; Griffin et al., 2017). Although clemizole is a first-generation anti-histamine, 49 other antihistamines from the screening database failed to exhibit anti-seizure activity suggesting, not unexpectedly, that antagonizing the H1 receptor does not decrease seizures in DS. Through a series of binding studies, it was discovered that clemizole binds serotonin 2 receptors. From additional blinded screening of targeted libraries two additional serotonin 2 receptor modulating drugs, lorcaserin and trazodone, were identified as effective in suppressing seizure activity in the *scn1lab*^s552^ larvae. Taken together, from our blinded, unbiased, screening of ~3,000 drugs, the three compounds which reduce seizures in *scn1lab*^s552^ larvae (clemizole, lorcaserin and trazodone) all bind serotonin 2 receptors. Furthermore, over 4% of all compounds tested are recognized to modulate serotonin signaling or bind serotonin receptors, suggesting this screening methodology can distinguish on-target effects of individual compounds (Figure 3). In addition, candidate screening of a selective serotonin reuptake inhibitor fenfluramine, which has shown some success as an add-on treatment for DS (Ceulemans et al., 2012) and is currently in Phase III clinical trials (https://clinicaltrials.gov/ct2/show/NCT02682927), also demonstrated efficacy against spontaneous seizures in the *scn1lab*^s552^ mutants (Dinday and Baraban, 2015; Sourbron et al., 2016). Although experimental evidence suggests that fenfluramine acts to modulate serotonergic signaling, Sourbron et al. (2017a) recently suggested that antagonism of the sigma-1 receptor is a putative mechanism of action for fenfluramine. Interestingly, retrospective analysis of our drug library database revealed eight sigma-1 receptor binding compounds that failed to suppress behavioral seizure activity in *scn1lab*^s552^ zebrafish using our blinded locomotion assay. As such, we could not independently confirm an antiepileptic action for sigma-1 antagonism.

Offering another alternative approach, Eimon et al. (2018) published a drug screening protocol using a multichannel local-field potential recording platform mimicking the invasive agarose-embedding procedure established in 2005 (Baraban et al., 2005). Here, *scn1lab*^s552^ zebrafish were tested at 7 dpf, but unlike previously discussed studies which focused on the naturally-occurring spontaneous unprovoked seizures, a 10 min light-provoked seizure protocol consisting of a dual-pulse light stimulus every 2 min was employed. Combined with an automated seizure detection algorithm to detect electrographic seizure-like events, 154 compounds were evaluated for their ability to restore provoked electrographic activity to a sibling level. Although swim behavior analysis identified stiripentol, diazepam, clonazepam and clobazam as decreasing light-provoked seizure-like activity, none of these drugs were successfully identified using the seizure algorithm or the behavioral analysis described by Eimon et al. (2018). For example, diazepam, clonazepam and clobazam (Onfi®), are the first line benzodiazepine AEDs used by DS patients, scored the same as the vehicle control using their LFP brain activity pattern algorithm combined with “deep behavioral phenotyping”. This suggests poor predictive validity of this assay to identify AEDs suitable for DS patients. Likewise, stiripentol (Diacomit®), an AED approved in Europe for the treatment of DS was also not identified. Furthermore, five compounds (with entirely unrelated mechanisms of action) that previously failed to show any antiepileptic efficacy against spontaneous seizures in *scn1lab*^s552^ zebrafish (Baraban et al., 2013; Dinday and Baraban, 2015; Griffin et al., 2017)—pargyline, progesterone, promethazine, mifepristone and fluoxetine—were identified as the “highest-ranked compounds” in this publication suggesting, again, that these assay outcomes should be interpreted with caution. Finally, although the LFP complexity scoring failed to successfully predict clemizole at 10 μM (a concentration some 10-fold lower than previously reported) using a provoked seizure assay, it was stated that “retesting clemizole at higher concentrations reduces the number of spontaneous seizures”, replicating our findings with *scn1lab*^s552^ zebrafish. Using an approach that does not first successfully identify AEDs clinically prescribed to DS patients makes it challenging to interpret the effectiveness of potential new therapies for this already difficult to treat patient cohort. Additionally, these studies highlight that drug discovery programs must consider the choice of model, as well as the predictability of the assay to have the best chance of identifying effective AEDs.

## Translating from the laboratory to the clinic

As alternative models like zebrafish emerge as valid preclinical models for drug discovery, understanding and translating pharmacokinetics remains to be fully explored. Pharmacokinetic ADME (absorption, distribution, metabolism, and excretion) studies represent a crucial aspect of drug development. These studies are traditionally performed in rodents and are not well-suited to zebrafish. In traditional mammalian models, drug pharamacokinetics can be easily established from administration of single or repeated drug concentrations. In zebrafish larvae, exposure to drug remains constant as the larvae is immersed in bathing media containing the drug, which is rapidly absorbed through the skin and gills. Currently, quantifying drug uptake into zebrafish larvae remains a limitation of this model. Differences in drug absorption are unavoidable and directly measuring drug concentrations in serum or tissues of microscopic larvae remains technically challenging particularly in a high-throughput drug screening environment. Currently, there are no zebrafish studies understanding how the effective concentrations in larvae can be related to appropriate effect levels in mammalian models, and we caution that these direct concentration comparisons may not be possible. As used, current zebrafish screening approaches can only assess whether a drug has anti-seizure properties (or not) and we would advise against over-interpreting concentrations used or ranking drugs based on effectiveness in these larval assays.

Nonetheless, with the current availability of accurate genetic models of DS and evaluating drugs against epilepsy, “personalized” treatment options are beginning to emerge. While this represents an exciting advance in the epilepsy field and an important alternative to traditional drug screening programs, the validity of drugs identified by these models will ultimately be determined in the clinic. Despite this potential, a major concern highlighted in several epilepsy community “white papers” has been the poor reproducibility of preclinical data for compounds progressing from academic laboratories to clinical trials (Galanopoulou et al., 2012, 2017; Simonato et al., 2014, 2017). As these reviews failed to adequately include or provide a rigorous evaluation of any preclinical zebrafish drug discovery research, we believe these types of concerns are irresponsibly premature and negligently misguided. In less than 5 years, using a well-characterized *scn1lab*^s552^ zebrafish model and pharmacologically validated methodologies described here, compounds effective against spontaneous seizures have already shown exciting early promise in clinical studies. As one small example, a serotonin receptor agonist (lorcaserin, Belviq®) identified only in a DS zebrafish model was used to treat five medically intractable DS patients and showed promising results in terms of reductions in seizure frequency and/or severity i.e., a 65% reduction in seizure frequency during the first 3-month treatment period (Griffin et al., 2017). Although this is the first “aquarium-to-bedside” example, and will ultimately require more rigorous clinical testing on larger patient cohorts, it hints at the tremendous potential a zebrafish-based platform holds for achieving true and effective personalized medicine. Furthermore, preclinical strategies that show “construct,” “face,” and “predictive” validity offer the best chance of success for identifying clinically effective treatments for genetic intractable epilepsy.

## Author contributions

AG and SB: manuscript preparation; AG, KH, and MA: locomotion screening and analysis; SH and SB: electrophysiology and analysis; LL and SB: experimental oversight and supervision.

### Conflict of interest statement

SB is a Co-Founder and member of the Scientific Advisory Board for Epygenix Therapeutics. The other authors declare that the research was conducted in the absence of any commercial or financial relationships that could be construed as a potential conflict of interest.
